# Identification and Validation of TYMS as a Potential Biomarker for Risk of Metastasis Development in Hepatocellular Carcinoma

**DOI:** 10.3389/fonc.2021.762821

**Published:** 2021-11-09

**Authors:** Shuai Li, Jingyuan Zhao, Linlin Lv, Deshi Dong

**Affiliations:** ^1^ Department of Pharmacy, The First Affiliated Hospital of Dalian Medical University, Dalian, China; ^2^ Regenerative Medicine Center, The First Affiliated Hospital of Dalian Medical University, Dalian, China

**Keywords:** hepatocellular carcinoma, metastasis, TYMS, prognostic biomarker, bioinformatics

## Abstract

Metastasis is the major cause of hepatocellular carcinoma (HCC) mortality. Unfortunately, there are few reports on effective biomarkers for HCC metastasis. This study aimed to discover potential key genes of HCC, which could provide new insights for HCC metastasis. GEO (Gene Expression Omnibus) microarray and TCGA (The Cancer Genome Atlas) datasets were integrated to screen for candidate genes involved in HCC metastasis. Differentially expressed genes (DEGs) were screened, and then we performed enrichment analysis of Gene Ontology (GO), together with Kyoto Encyclopedia of Genes and Genomes (KEGG). A protein-protein interaction network was then built and analyzed utilizing STRING and Cytoscape, followed by the identification of 10 hub genes by cytoHubba. Four genes were associated with survival, their prognostic value was verified by prognostic signature analysis. Thymidylate synthase (TYMS) gene was identified as significant HCC metastasis-associated genes after mRNA expression validation and IHC analysis. TYMS silencing in HCC cells remarkedly inhibited growth and invasion. Finally, we found TYMS silencing dramatically decrease DNA synthesis and extracellular matrix (ECM) degradation, resulting in the inhibition of HCC metastasis, indicating TYMS had close associations with HCC development. These findings provided new insights into HCC metastasis and identified candidate gene prognosis signatures for HCC metastasis.

## Introduction

Hepatocellular carcinoma (HCC) is the fourth most common cause of cancer-related deaths in the world (1). The 5-year overall survival remains relatively poor, because of the difficulty associated with the early diagnosis and metastasis (2). Metastasis is responsible for as much as 90% of most cancer-related deaths (3). The multistep process of invasion and metastasis has been schematized as a sequence of discrete steps, often termed the invasion-metastasis cascade (4). The acquisitions of extensive invasion potencies by cancer cells are key components in the metastatic cascade. To enter the blood or lymphatic circulation, carcinoma cells must first degrade the ECM to break tissue barriers, which is considered a key step promoting tumor invasion and metastasis (5). Therefore, it is essential to elucidate the molecular mechanism of HCC metastasis and identify potential molecular biomarkers for prognosis prediction.

Along with the development of microarray and high-throughput sequencing technology, many tumor prognostic markers were reported by using GEO and TCGA. Databases based on the TCGA data set (GEPIA, cBioPortal, Human Protein Atlas, etc.) can analyze and confirm the expression of hub genes and the impact on survival. These identified genes are involved in the proliferation and metastasis of HCC, thereby affecting the survival and prognosis of HCC patients. The invasiveness and metastasis of HCC severely limit the improvement of the curative effect of HCC. Therefore, excavation of the genes involved in metastasis has been an important goal for the past several decades. Although many biomarkers for HCC have been identified, there are few reports on biomarkers for HCC metastasis. TYMS is an enzyme that catalyzes the conversion of dUMP to dTMP, and is the main intracellular target of the active 5-FU metabolite (6). There are many studies in the literature demonstrating prognostic and predictive values of TYMS presence in cancer (6–14), however, its role as a marker of HCC metastasis is not known.

In this study, the differential mRNA expression data of HCC from the TCGA and GEO databases were analyzed to identify key genes. The integrated bioinformatics analysis by investigating the functions and pathways of the gene was used to further investigated their potentiality of being biomarkers in HCC. Functional enrichment and protein interaction (PPI) analysis combined with survival analysis were used to further screen genes that are critical for HCC development. The prognostic value of the genes was evaluated using the ROC curve (Receiver Operating Characteristic Curve) and survival analysis. *In vitro* experiment was conducted to elaborate the potential roles of the biomarker in the proliferation and invasion of HCC cells.

## Materials And Methods

### Data Collection and Data Preprocessing

The gene expression profiling datasets (GSE28248 and GSE27635) were obtained from the GEO database (https://www.ncbi.nlm.nih.gov/geo/). The dataset for GSE28248 includes intratumoral and peritumoral tissues of 20 paired HCC samples patients with lymph node-positive. GSE27635 incorporates 24 pairs of intratumoral and peritumoral tissue from HCC patients with bone metastases. TCGA HCC dataset, coupled with the clinical data, includes 50 normal and 369 tumor samples.

### Screening of DEGs

To investigate DEGs among normal and tumor samples, the limma package in R was used with the cut‐off criteria of |log 2‐fold‐change [FC]| > 1 and a corrected p < 0.05. The common DEGs in GSE28248 and TCGA were screened for subsequent analysis.

### Gene Ontology and KEGG Pathway Analysis

Gene ontology (GO) term enrichment analysis and Kyoto Encyclopedia of Genes and Genomes (KEGG) pathway analysis were performed by using DAVID (https://david.ncifcrf.gov/). The KEGG pathway and GO enrichment analyses of upregulated and downregulated genes were based on the threshold of adjusted p‐value < 0.05.

### Protein-Protein Interaction (PPI) Network Construction and Hub Genes Identification

To evaluate the interrelationships behind the list of genes, we used the STRING database for analysis. The Cytoscape software for visualizing and analyzing molecular interaction networks, was used to build a PPI network. Hub genes were selected from genes intersection calculated by using the Cytoscape plugin cytoHubba.

### Risk Stratification (RS) and ROC Curves

Multivariate Cox analysis was further used to determine whether the four prognostic genes were independent prognosis factors of the patient’s OS (Overall survival). The risk score model was constructed based on a linear integration of the expression level multiplied regression model (β) using the following formula: Risk score= expRNA1×βRNA1 + expRNA2×βRNA2+…+expRNAn×βRNAn. The sensitivity and specificity of the risk model were calculated by the AUC (Area under the curve) of the ROC curve with the “survival ROC” R package.

### Validation of the Key Genes Based on TCGA Data and HPA Database

Gene Expression Profiling Interactive Analysis (GEPIA: http://gepia.cancer‐pku.cn/), a web‐based tool to display the survival and expression patterns between tumor and normal groups was used to verify our results. Furthermore, the validation of the protein levels of the key genes was carried out using the Human Protein Atlas (HPA) database (https://www.proteinatlas.org/).

### Validation of the TYMS in Clinical Tissue Samples

A total of 24 paired HCC patients specimens (tumor and adjacent nontumor tissues) were collected from the Biobank of the First Affiliated Hospital of Dalian Medical University (Liaoning, China). The samples were removed from hospitalized patients at the Department of Hepatobiliary Surgery, First Affiliated Hospital of Dalian Medical University (Liaoning, China) from 2018 to 2021. Samples used in this study were approved by the Committees for Ethical Review of Research at the First Affiliated Hospital of Dalian Medical University. The TYMS gene mRNA expression between tumor and normal groups was assayed by real-time PCR.

### Cell Culture and Cell Transfection

One immortalized normal liver cell line L02 and three human HCC cell line MHCC-97H, BEL-7402 and HuH-7 were obtained from the Chinese Type Culture Collection, Chinese Academy of Sciences. All cells were cultured in DMEM supplemented with 10% fetal bovine serum and kept in a humidified atmosphere of 5% CO_2_ at 37◦C. In order to inhibit the expression of TYMS, we transfected two small interfering RNAs (siRNAs) targeting the TYMS coding sequence. The siRNAs were purchased from GenePharma (Shanghai, China) and sequences are as follows: 1. GGAGTTGACCAACTGCAAA 2. CAACCCTGACGACAGAAGA. The efficiency of infection was assayed by real-time PCR.

### Quantitative Real-Time PCR

Total RNA was isolated using Trizol reagent (Thermo Fisher) and reverse-transcribed according to the manufacturer’s instructions. The cDNA was subjected to quantitative Real-Time PCR using the SYBR Green PCR Master Mix (Thermo Fisher) and the assay was performed on a 7500 fast Real-Time PCR system (Applied Biosystems by Life Technologies). Relative expression levels were normalized to GAPDH. The 2^−ΔΔCt^ method was used to compare the fold differences in expression. Primer sequences were listed as follows: TYMS forward 5’- TGGGGCAGAATACAGAGATATGG-3’ and reverse 5’- TGATGGTGTCAATCACTCTTTGC-3’, GAPDH forward 5’- ACAACTTTGGTATCGTGGAAGG-3’ and reverse 5’- GCCATCACGCCACAGTTTC-3’.

### Western Blotting

The TYMS (#15047-1-AP) and GAPDH (#10494-1-AP) antibodies were purchased from Proteintech. Proteins were electrophoresed in SDS-PAGE gel and transferred onto PVDF membranes. The membranes were blocked with 5% skim milk in TBS/0.1% Tween 20 containing for 0.5 h and then incubated overnight with the primary antibodies at 4°C. The membrane was further incubated with HRP-conjugated secondary antibodies for 1 hours at room temperature. Protein bands were visualized with ECL substrates.

### Cell Proliferation

Viable cells were measured using the Cell Counting Kit-8 (CCK-8) proliferation assay (APExBIO) according to the manufacturer’s guidelines.

### Colony Formation

For colony formation assay, 500 cells were plated in 6-well plates and culture for 14 days. Cells were stained with 0.1% crystal violet and the number of colonies was counted.

### EdU (5-Ethynyl-2’-Deoxyuridine) Incorporation Assay

Cells were incubated with 10 μM EdU (APExBIO) for 2 h at 37°C. After incubation, the cells were washed with PBS and fixed with 4% paraformaldehyde (PFA) for 15 min and followed by incubation with 2.5% Triton X-100 for 10 min at room temperature. Cells were washed and a 100 µl Click-iT reaction cocktail was added to the sample incubated for 30 min at room temperature. 10 μg/ml hoechst33342 was added and incubated for 10 min at room temperature.

### Wound Healing Assay

Cells were cultured in a 6-well plate until they reached 70–80% confluence. A scratch was made through the cell monolayer using a sterile pipette tip. The cells were allowed to migrate into the wound area for 48 h. The percent change in migration was determined by using ImageJ.

### Transwell Invasion Assay

Cell invasion was performed using transwell chambers (8-µm pore size, Corning). For invasion assays, 1.0 × 10^5^ cells were seeded into upper inserts with a Matrigel-coated membrane. The cells were allowed to migrate or invasion for 24 h. The cells remaining on the upper membrane were removed with cotton wool. The remaining cells were fixed, stained with crystal violet, and analyzed by inverted microscopy.

### Matrix Degradation Assay

In brief, glass coverslips were coated with 0.2 mg/ml FITC-gelatin (Anaspec), cross-linked with 0.5% glutaraldehyde, and then treated with 5mg/ml sodium borohydride for 5 min. Cells were plated on FITC-gelatin for 8 h and fixed with 4% formaldehyde and analyzed for gelatin-degradation under a fluorescence microscope (Leica). Gelatin-degradation spots were quantified using ImageJ.

### Statistical Analyses

Results are recorded as means ± standard error of the mean for at least three independent experiments, and analyzed by one-way ANOVA, two-tailed unpaired Student’s t-test, or Mann-Whitney test. Statistical analyses were performed using the GraphPad Prism 6 software. Data were considered statistically significant as follows: *P< 0.05, **P< 0.01, and ***P< 0.001.

## Results

### Identification of DEGs and Functional Annotation

**Figure 1:**
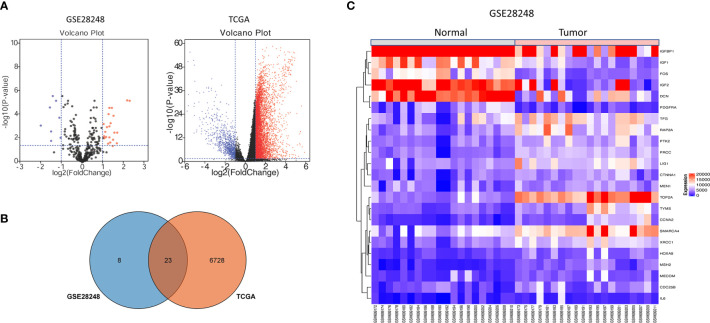
DEGs identification from GEO and TCGA. **(A)** Volcano plot visualizing DEGs in GSE28248. **(B)** Overlapping DEGs between GEO and TCGA database. **(C)** The expression heatmap of the overlapping DEGs in GSE28248.

The gene expression profiling of GSE28248, which contained 20 pairs of HCC patients with bone metastases samples and normal samples, was analyzed with GEO2R. A total of 31DEGs were identified (23 upregulated and 8 downregulated genes were listed in **Supplementary Table 1**). The volcano plot of GSE28248 and TCGA DEGs is shown in **Figure 1A**. To confirm the reliability of DEGs in HCC, we obtained overlapping DEGs (**Figure 1B**) of the two datasets including 16 upregulated and 7 downregulated genes. The heatmap of the top DEGs is shown in **Figure 1C**.

**Figure 2:**
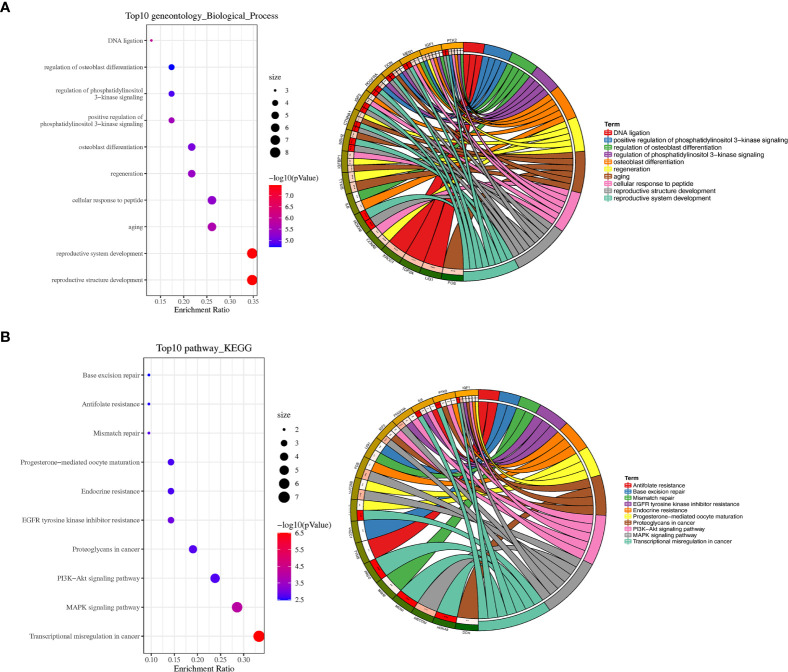
GO and KEGG pathway enrichment analyses. **(A)** Bubble plot of biological processes enrichment analysis of DEGs and circle plot of biological processes enrichment analysis of DEGs. **(B)** Bubble plot of KEGG pathway enrichment analysis of DEGs and circle plot of KEGG pathway enrichment analysis of DEGs. *P < 0.05, **P < 0.01, and ***P < 0.001.

To further analyze the biological function of the DEGs, we processed GO function and KEGG pathway enrichment analysis with DAVID. With GO function analysis, we discovered that the DEGs are mostly enriched in the biological process of regulation of aging, regulation of phosphatidylinositol 3−kinase signaling (**Figure 2A**). As for molecular function, protein N−terminus binding and growth factor activity are contained in the top 10 enrichment classes (**Supplementary Figure 1A**). As we conducted the KEGG pathway analysis, we found that these DEGs are mainly enriched in the MAPK signaling pathway and PI3K−Akt signaling pathway (**Figure 2B**).

### PPI Network Analysis and Screening for Hub Genes

**Figure 3:**
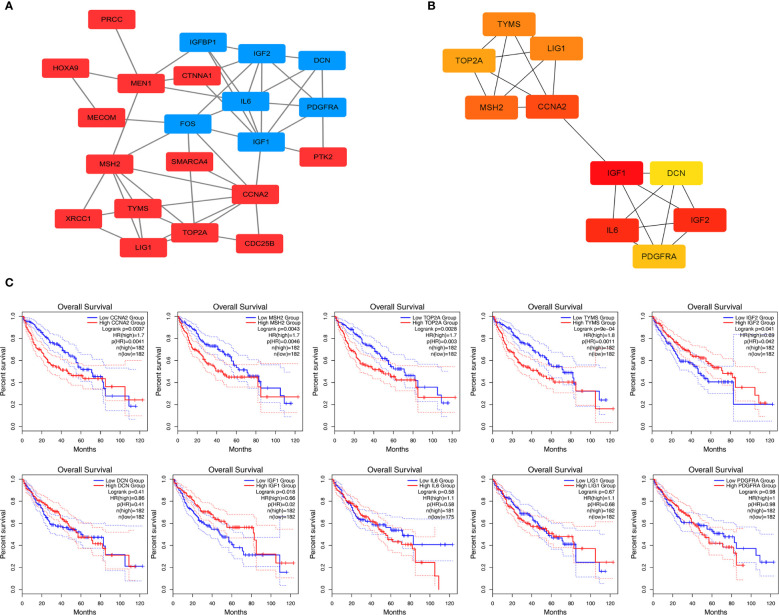
Protein-protein interaction network and overall survival analysis of DEGs. **(A)** Based on the STRING online database, a PPI network was constructed containing 23 DEGs (upregulated DEGs labeled in red and downregulated DEGs labeled in blue). **(B)** Identification of hub genes MCC algorithm. **(C)** Survival analysis for TYMS in HCC from the GEPIA2 database.

STRING is an online tool for predicting protein-protein interactions (PPI) (15). After importing the overlapped DEGs into the online tool STRING, we obtained the PPI network of these genes (**Figure 3A**). Overlapped DEGs were imported into Cytoscape to screen the hub genes inside the network with Maximal Clique Centrality (MCC) algorithm. The MCC of each node was calculated by CytoHubba, a plugin in Cytoscape. In this study, the genes with the top 10 MCC values were considered as hub genes (**Figure 3B**).

### Verification of the Prognostic Values of Hub Genes

We used GEPIA to evaluate the contribution of the 10 hub genes to clinical outcomes. The results demonstrated four genes CCNA2 (Cyclin-A2), MSH2 (MutS homolog 2), TOP2A (Topoisomerase 2-alpha) and TYMS were significantly positively correlated to patient outcomes, IGF2 was negatively correlated to patient outcomes (**Figure 3C**). The remaining hub gene survival analyses did not show statistical significance.

**Figure 4:**
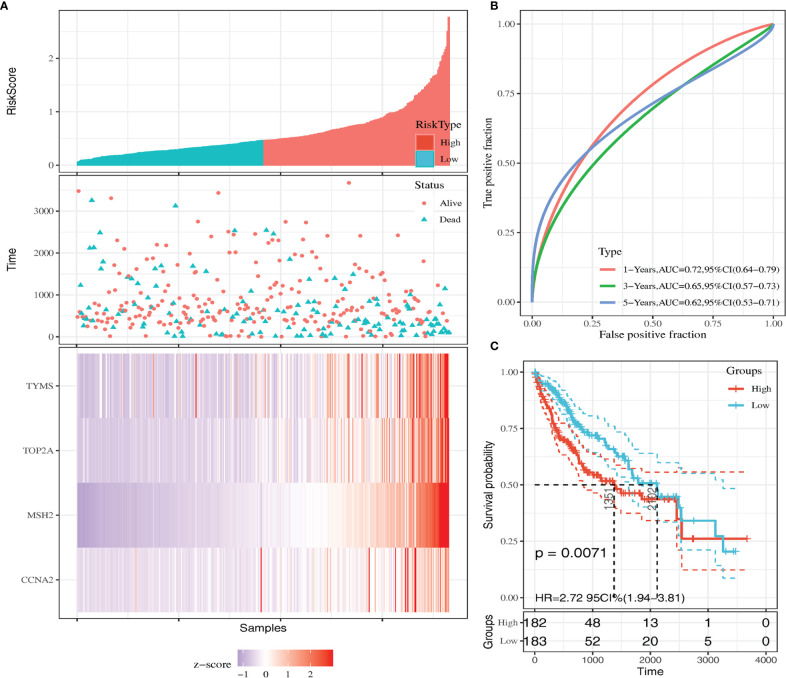
Kaplan-Meier survival curve of overall survival between the high‐risk group and low‐risk group **(A)** Risk score, survival time, survival status, and expression of the four genes signature. **(B)** AUC for the four genes classification. **(C)** Kaplan-Meier survival curve of the four genes from the TCGA dataset.

We calculated the risk score for each sample using the Sanger box website and used the cut-off values of the training set to classify the samples into high and low risk groups in TCGA HCC data. The distribution of risk score, survival status, and the expression of four genes for each patient were analyzed (**Figure 4A**). With the increase in riskscore, the expression levels of CCNA2, MSH2, TOP2A and TYMS showed an increasing trend. The AUC of the 1-, 3-, and 5-year ROC curve were 0.72, 0.65, and 0.62 (**Figure 4B**). Kaplan–Meier curve showed that the overall survival time of patients in the low‐risk group was significantly longer than in the high‐risk group (**Figure 4C**).

### Validation of Genes Expressions

**Figure 5:**
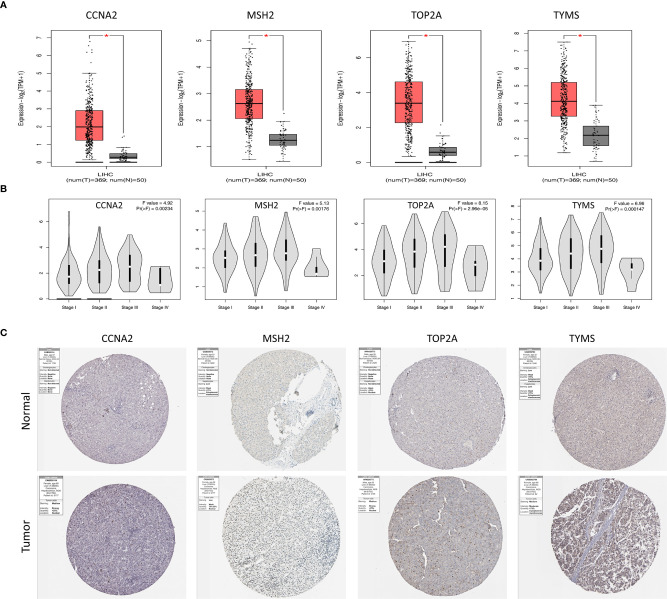
Validation of genes expressions of four hub genes. **(A)** Box plots of CCNA2, MSH2, TOP2A and TYMS expression in TCGA tumor and normal tissues. **(B)** Correlations between differently expressed genes and the pathological TNM stage of HCC patients. **(C)** Representative immunohistochemistry staining of CCNA2, MSH2, TOP2A and TYMS in HCC tissue obtained from the Human Protein Atlas. *P < 0.05.

Validation of the expression of four prognosis-related genes with GEPIA, we detected the mRNA levels of four genes in TCGA database. CCNA2, MSH2, TOP2A and TYMS were found to be highly expressed in tumor tissues compared with normal tissues (**Figure 5A**). The significant correlation between four genes and the stage was also verified using the GEPIA (**Figure 5B**), they might be an oncogene for HCC that participates in tumorigenesis.

The protein expression of the genes was determined using immunohistochemistry (IHC) from the Human Protein Atlas database (HPA) to verify the transcriptome analysis results. The protein expression levels of TYMS showed upregulated (**Figure 5C**).

### Validation in Clinical Tissue Samples

**Figure 6:**
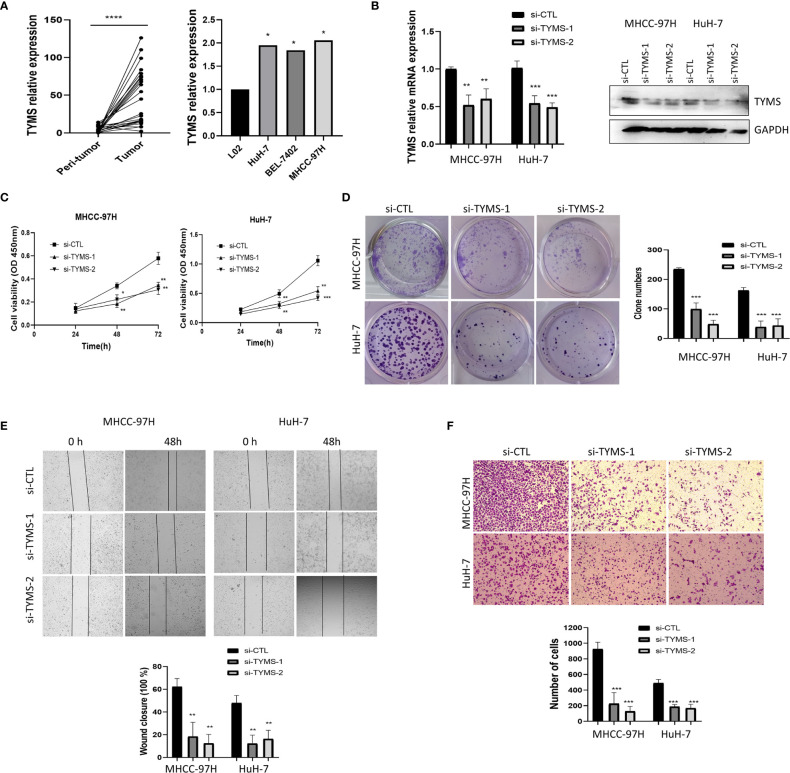
TYMS affect cell growth and invasion in MHCC-97H and HuH-7 cells. **(A)** The mRNA expression of TYMS in 24 pairs of HCC tissues and HCC cell lines. **(B) **The expression of TYMS in MHCC-97H and HuH-7 cells. The expression of TYMS was detected by qPCR and western blot. The 2^−ΔΔCt^ method was used to quantify the mRNA fold changes, GAPDH was used as the internal control. **(C)** CCK8 assay was used to explore the effect of TYMS expression on cell viability. **(D)** Representative images and quantification of colony formation assay. The number of colonies with > 50 cells was scored after 14 days of incubation. **(E)** wound-healing analysis and **(F)** Transwell invasion assays represented the migration and invasion capacity of HCC cells. *P < 0.05, **P < 0.01, ***P < 0.001 and ****p < 0.0001.

In order to validate the bioinformatics analysis results, 24 paired HCC tumor and peri-tumor samples were studied. Comparing with peri-tumor controls, the expression of TYMS was significantly increased in HCC tissues (**Figure 6A**) which were consistent with the bioinformatics results obtained by the TCGA dataset. We studied the expression of TYMS in three HCC cell lines and one normal liver cell line (L02). Consistent with the results in cancer tissues, we observed that TYMS expression levels were higher in the liver cancer cell lines than in the normal liver cell line (**Figure 6A**).

### TYMS Promotes HCC Cells Proliferation and Invasion

To analyze the impacts of TYMS on cancer cell proliferation and invasion. Two siRNA targeting TYMS were transfected into MHCC-97H and HuH-7. The silencing efficiency was verified by real-time PCR and western blot (**Figure 6B**). We performed a cell counting kit-8 assay to assess proliferation, the results showed that the downregulation of TYMS significantly decreased MHCC-97H and HuH-7 cells viability to a significant extent (**Figure 6C**). Colony formation assays found silencing of TYMS expression displayed significant differences in colony formation comparing with the control (**Figure 6D**), indicating that TYMS affects tumor growth.

To determine whether TYMS had a role in HCC cells invasion, we first investigated the effect of TYMS on migration. Wound healing assay was performed, the results suggested that TYMS promoted migration of MHCC-97H and HuH-7 cells (**Figure 6E**). Cells were subjected to chemotaxis invasion assays by seeding at equal numbers in a transwell invasion chamber with Matrigel. The invasion capabilities of MHCC-97H and HuH-7 cells were reduced by inhibition of TYMS expression compared with the control cells (**Figure 6F**). These findings suggest that TYMS promotes migration and invasion in HCC cells.

### TYMS Promotes HCC Cells DNA Synthesis and Extracellular Matrix Degradation

**Figure 7:**
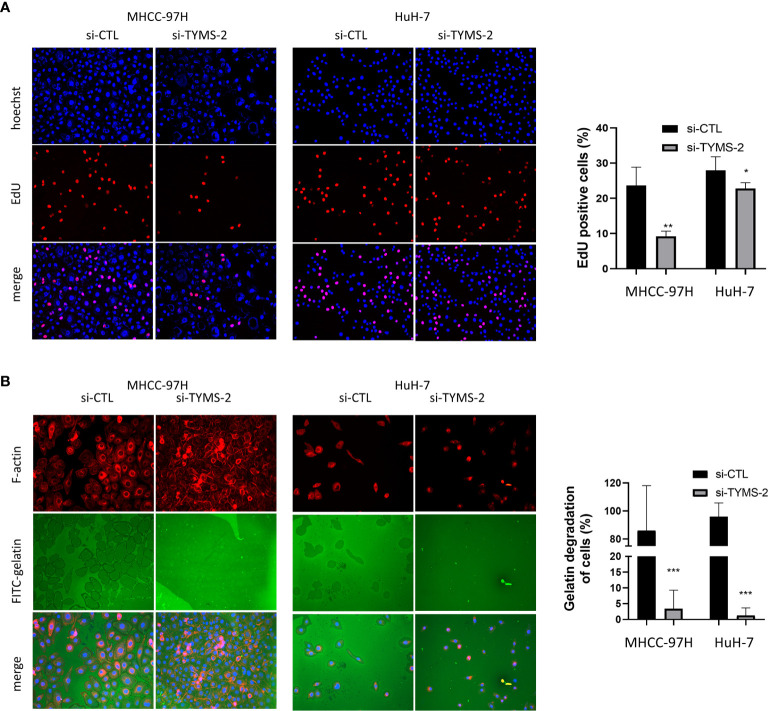
TYMS plays a role in HCC cell’s DNA synthesis and invasiveness. **(A)** The EdU incorporation analyses the effects of TYMS on cell proliferation. **(B)** TYMS is necessary for ECM degradation in MHCC-97H and HuH-7 cells. Cells were plated on coverslips coated with FITC- gelatin (green) for 8 h, F-actin was stained with phalloidin (red), and nuclei were stained with DAPI (blue). Gelatin degradation was shown as black areas. *P < 0.05, **P < 0.01, and ***P < 0.001.

EdU is a nucleoside analog of thymidine and is incorporated into DNA during active DNA synthesis (16). To further confirm the effect of TYMS on cell proliferation, we assessed DNA synthesis using an EdU incorporation assay. The results showed that TYMS had an effect on the DNA synthesis of MHCC-97H and HuH-7 cells (**Figure 7A**). These results indicate that TYMS affects the proliferation of HCC cells.

Metastasis is a highly inefficient process, and certain steps of the invasion-metastasis cascade are extraordinarily inefficient (5). ECM degradation is an early and essential step in cancer metastasis, which is a key factor for determining patient survival in different types of solid tumors (3). To dissect the cellular functions of TYMS in promoting HCC cells invasion, we examined whether expression of TYMS was associated with ECM degradation. MHCC-97H and HuH-7 cells were plated on FITC–labeled gelatin matrix to assess their abilities to degrade the matrix. TYMS knockdown resulted in a potent reduction in matrix degradation in both cells (**Figure 7B**). These results indicate TYMS is necessary for extracellular matrix degradation in HCC cells.

## Discussion

In this study, the genes potentially affecting metastasis in HCC were identified based on two integrated GEO and TCGA HCC data sets. DEGs were screened out to investigate function and pathways enrichment. Enrichment analysis showed that the DEGs were significantly enriched in terms of MAPK signaling pathway and PI3K−Akt signaling pathway. The expressions of CCNA2, MSH2, TOP2A and TYMS mRNA were significantly upregulated in HCC. A combination of the identified genes was used as an indicator for risk stratification that helps to predict the prognosis of HCC. The overall survival rate indicated, HCC patients with high expression of CCNA2, MSH2, TOP2A and TYMS showed poor overall survival.

CCNA2 plays a critical role in the control of cell cycle transitions and has a potential role in tumorigenesis (17). CCNA2 belongs to the highly conserved cyclin family and is expressed in almost all tissues in the human body (18). The MSH2 gene is responsible for recognizing nucleotide mismatches occurring during DNA replication (19), particularly in the case of microsatellite instability (20). TOP2A is a key enzyme in DNA replication and transcription (21). TOP2A expression is detected in various types of tumors (22), it is typically expressed at high levels in rapidly proliferating cells (23). TYMS is a highly conserved enzyme and essential for cell survival due to its important role in DNA biosynthesis (24–26). TYMS is also an mRNA-binding protein by binding mRNA to coordinately regulate the cellular gene expression (8). TYMS may regulate several key aspects of cell cycle control, including apoptosis and chemosensitivity (8). The high level of TYMS is conducive to the development of cancer, so it is considered to be an oncogene. TYMS is one of the most relevant targets for anti-tumor therapy, a lot of work has been done on TYMS as an anti-tumor target and drug resistance in the past (27–30). IHC staining obtained from the Human Protein Atlas database showed that the expression of TYMS was at high levels consistent with mRNA expression levels. The transcription is associated with promoter

methylation (31), to discover the reason for the upregulation of the TYMS gene in HCC, TCGA database was used to analyze the expression of TYMS and its methylation status. We found that the methylation level of TYMS was negatively correlated with the transcription level (**Supplementary Figure 1B**), and its low methylation status in HCC may have contributed to the up-regulated expression. The gene expression profiling also proved that TYMS is up-regulated in metastatic tumor tissues from GSE27635 (**Supplementary Figure 1C**), which contained 24 pairs of intratumoral and peritumoral tissue from HCC patients with bone metastases. Compare with microarrays, high-throughput sequencing offers higher accuracy and a larger dynamic range (32). Therefore, the TCGA HCC dataset using high-throughput sequencing technology has much more DEGs compare with GSE28248 and GSE27635 using microarrays. Therefore, the GSE28248 and GSE27635 used in the study may limit the discovery of more HCC metastasis biomarkers and also affect the further verification of biomarkers.

Although a large number of studies have focused on the association of TYMS with carcinogenesis, prognosis, chemotherapy reaction in colorectal cancer (13, 14), ovarian cancer (10), pancreatic cancer (11), cervical cancer (12), and breast cancer (9), few studies focused on the role of TYMS in metastatic HCC patients. More in-depth studies on molecular function and clinical relevance are needed to better understand the role of TYMS in promoting HCC progression and metastasis. Combined with bioinformatics and *vitro* experiments, we analyzed the gene changes and the role of TYMS in HCC. The downregulation of TYMS could reduce the proliferation and invasion of HCC cells. Colony formation assay indicated that the downregulation of TYMS could reduce the proliferation ability of HCC cells. It is possible that TYMS affects single cell proliferation ability. TYMS induces ECM degradation and invasion of HCC cells, which plays an active role in the metastatic progression of HCC. Results in the present work may reflect the role of TYMS in HCC metastasis and this raises the possibility to assess the clinical association of TYMS and metastasis of HCC. Detection of the TYMS expression in tumor cells is expected to make an early diagnosis of HCC metastasis. Our results provide new opportunities for the treatment of HCC metastasis targeting TYMS. In addition, in the follow-up work, a more detailed molecular mechanism analysis of TYMS is needed to clarify its role in promoting the metastasis of HCC.

As summarized in our work by bioinformatics analysis and *in vitro* experiment, we provided the evidence that TYMS is abnormally expressed in metastatic HCC, TYMS can be used as an indicator for risk stratification that helps to predict the prognosis of HCC. TYMS promotes cell DNA synthesis and ECM degradation, it has an impact on the proliferation and invasion of HCC cells. As a prognostic biomarker, TYMS might provide essential information regarding personalized treatment decisions for individual patients and improve the therapeutic gain. It deserves more exploration and demonstration for its potentiality in diagnosis, prognosis, and therapeutic target.

## Data Availability Statement

The original contributions presented in the study are included in the article/**Supplementary Material**. Further inquiries can be directed to the corresponding author.

## Ethics Statement

The studies involving human participants were reviewed and approved by Ethical Committee of First Affiliated Hospital of Dalian Medical University.

## Author Contributions

DD conceived and supervised the study. SL designed experiments. SL, JZ, and LL performed experiments. DD provided new tools and reagents. SL analyzed data. SL wrote the manuscript. DD made manuscript revisions. All authors contributed to the article and approved the submitted version.

## Funding

This work is supported by a grant from the Natural Science Foundation of China (No. 62072070); Dalian Medical Science Research Project from the Dalian Municipal Health Commission (No. 2012006).

## Conflict of Interest

The authors declare that the research was conducted in the absence of any commercial or financial relationships that could be construed as a potential conflict of interest.

## Publisher’s Note

All claims expressed in this article are solely those of the authors and do not necessarily represent those of their affiliated organizations, or those of the publisher, the editors and the reviewers. Any product that may be evaluated in this article, or claim that may be made by its manufacturer, is not guaranteed or endorsed by the publisher.
