# Supplementary material for: Identification and Validation of TYMS as a Potential Biomarker for Risk of Metastasis Development in Hepatocellular Carcinoma
Source: Front Oncol. 2021 Nov 9;11:762821. doi: 10.3389/fonc.2021.762821 (PMC8630669; doi:10.3389/fonc.2021.762821)
Supplement: Supplementary file 1 [file DataSheet_1.docx]

Supplementary TABLE 1 | List of 31 DEGs identified in GSE28248

| Name | Expression |
| --- | --- |
| IGF2 | down-regulated |
| DCN | down-regulated |
| PDGFRA | down-regulated |
| BCL2 | down-regulated |
| FOS | down-regulated |
| IGF1 | down-regulated |
| IGFBP1 | down-regulated |
| TNFRSF10A | down-regulated |
| LIG1 | up-regulated |
| XPA | up-regulated |
| RAD52 | up-regulated |
| MECOM | up-regulated |
| PTK2 | up-regulated |
| CASP3 | up-regulated |
| ETV6 | up-regulated |
| HOXA9 | up-regulated |
| RBBP5 | up-regulated |
| CDC25B | up-regulated |
| RAP2A | up-regulated |
| CTNNA1 | up-regulated |
| SMARCA4 | up-regulated |
| TYMS | up-regulated |
| MEN1 | up-regulated |
| NOTCH1 | up-regulated |
| XRCC1 | up-regulated |
| PRCC | up-regulated |
| IL6 | up-regulated |
| TFG | up-regulated |
| TOP2A | up-regulated |
| CCNA2 | up-regulated |
| MSH2 | up-regulated |


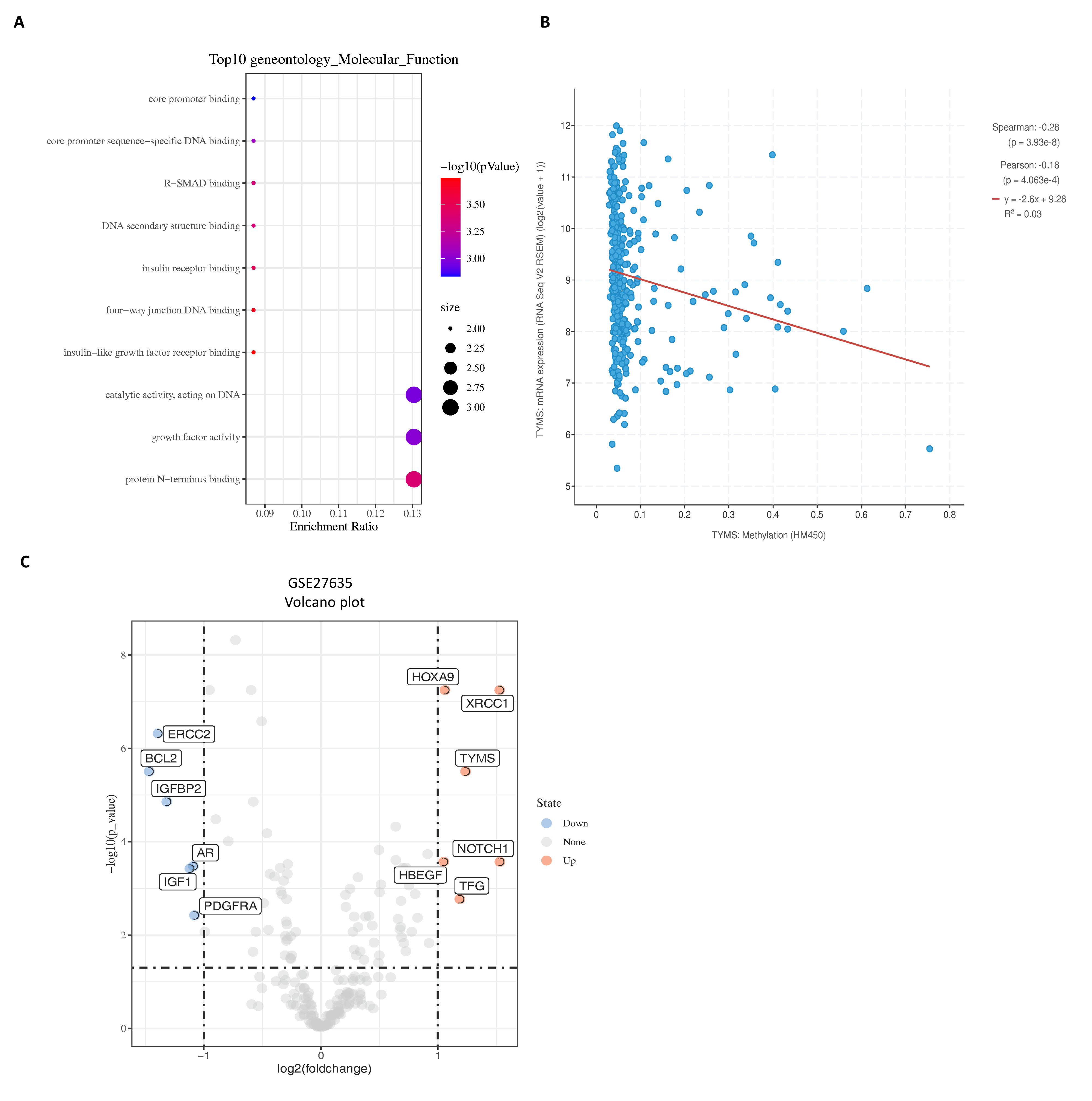


Supplementary FIGURE S1| (A) Bubble plot of molecular function enrichment analysis of DEGs. (B) The relationship between TYMS methylation and expression in TCGA HCC dataset. (C)Volcano plot visualizing DEGs in GSE27635.
